# The SAFE-T upper endoscopy tool: a web-based application for the point-of-care evaluation of gastroenterology fellow performance in upper endoscopy

**DOI:** 10.1093/gastro/goaa031

**Published:** 2020-07-29

**Authors:** Navin L Kumar, Guillaume Kugener, Kelly E Hathorn, Molly L Perencevich, Kunal Jajoo, John R Saltzman

**Affiliations:** 1 Division of Gastroenterology, Hepatology and Endoscopy, Brigham and Women’s Hospital, Boston, MA, USA; 2 Harvard Medical School, Boston, MA, USA; 3 Massachusetts Institute of Technology, Cambridge, MA, USA

**Keywords:** endoscopy, education, assessment, tool, fellowship, trainee

## Abstract

**Background:**

Attending assessment is a critical part of endoscopic education for gastroenterology fellows. The aim of this study was to develop and validate a concise, web-based assessment tool to evaluate real-time fellow performance in upper endoscopy.

**Methods:**

We developed the Skill Assessment in Fellow Endoscopy Training (SAFE-T) upper endoscopy tool to capture both summative and formative feedback in a concise, five-part questionnaire. The tool mirrors the previously validated SAFE-T colonoscopy tool and is administered electronically via a web-based application. We evaluated the tool in a prospective study of 15 gastroenterology fellows (5 fellows each from Years 1–3 of training) over the 2018–2019 academic year. An independent reviewer evaluated a subset of these procedures and completed both the SAFE-T and Assessment of Competency in Endoscopy (ACE) upper endoscopy forms for reliability testing.

**Results:**

Twenty faculty completed 413 SAFE-T evaluations of the 15 fellows in the study. The mean SAFE-T overall score differentiated each sequential fellow year of training, with first-year cases having lower performance than second-year cases (3.31 vs 4.25, *P *<* *0.001) and second-year cases having lower performance than third-year cases (4.25 vs 4.56, *P *<* *0.001). The mean SAFE-T overall score decreased with increasing case-complexity score, with straightforward compared with average cases (3.98 vs 3.39, *P *<* *0.001) and average compared with challenging cases (3.39 vs 2.84, *P *=* *0.042). In dual-observed procedures, the SAFE-T tool showed excellent inter-rater reliability with a Kappa agreement statistic of 0.815 (*P *=* *0.001). The SAFE-T overall score also highly correlated with the ACE upper endoscopy overall hands-on score (*r *=* *0.76, *P *=* *0.011).

**Conclusions:**

We developed and validated the SAFE-T upper endoscopy tool—a concise and web-based means of assessing real-time gastroenterology fellow performance in upper endoscopy.

## Introduction

Learning how to perform endoscopy is a key objective of gastroenterology (GI) fellowship programs [1]. Although simulation is becoming an increasing part of this education process, the majority of learning occurs through the ‘apprenticeship’ model, in which fellows perform endoscopic procedures on patients under the supervision of an attending gastroenterologist [2, 3]. As part of this model of teaching, attending assessment and delivery of feedback plays a critical role in fellow skill development. Such feedback is most effective when delivered immediately after the observed teaching encounter [4].

A handful of assessment tools for upper endoscopy have been developed to help faculty to assess fellow endoscopic skills and guide the delivery of feedback [5–7]. Of these tools, the Global Assessment of Gastrointestinal Endoscopic Skills in Upper Endoscopy (GAGES-UE) is the most concise but lacks any information on cognitive skills or more advanced interventions performed in therapeutic upper endoscopy [5]. The Assessment of Competency in Endoscopy (ACE) and Direct Observation of Procedural Skills (DOPS) for upper endoscopy forms are more comprehensive but as a result have multiple questions to complete (12–13 questions for the ACE form depending on interventions performed; 34 questions for the DOPS form), which limits their use as continuous assessment tools in everyday clinical practice [6, 7]. Notably, a recent nationwide survey showed that only a minority of fellowship programs (30%) reported using any form of validated assessment [3].

To improve the continuous assessment of fellow endoscopic performance, we recently developed and provided validity evidence for the Skill Assessment in Fellow Training (SAFE-T) tool for colonoscopy [8]. This evaluation tool contains five questions to complete (including both summative and formative assessment) and is administered via a web-based application that is optimized for smartphone use. The SAFE-T overall score was able to differentiate fellow performance by trainee year, case complexity, and cecal intubation, and showed excellent inter-rater reliability in dual-observed procedures. Surveyed faculty found the smartphone application easy to use and many remarked that using the tool reminded them to provide verbal feedback to their fellows at the end of each case. In this initial study, we focused on colonoscopy assessment and did not assess upper endoscopy fellow performance.

Our aim was to develop and validate a concise and analogous SAFE-T upper endoscopy tool that included both summative and formative feedback and utilized a web-based application to facilitate its use as a point-of-care evaluation tool for GI fellow endoscopic performance.

## Materials and methods

### Assessment tool design

We developed a five-part questionnaire specific to upper endoscopy training based on the framework of the SAFE-T colonoscopy tool (Table 1). The first question assesses objective performance by the fellow during the procedure, as determined by the farthest landmark reached without any hands-on assistance from the attending gastroenterologist. The second question gathers information on any interventions performed and informs the third question on case complexity, which is assessed on a three-point scale with anchors based on the type of intervention performed (if any) and the location of the intervention (accessible or difficult location). The fourth question requests faculty to rate the overall performance of the fellow on a five-point scale from beginner to superior with anchors based on the degree of hands-on assistance and coaching, as well as efficiency in completing the procedure. This summative form of assessment is identical to the five-point scale used in the SAFE-T colonoscopy tool. The fifth and final question of the SAFE-T tool captures formative feedback for the fellow as a single area to improve for the next case. This question contains 18 different skills for the faculty member to choose from as well as an option for no specific area to improve. The 18 skills include both cognitive and motor tasks critical to the successful completion of an upper endoscopy, such as pre-procedure evaluation, safe endoscopic advancement, pathology identification, and performance of specific interventions. We identified these skills based on a literature review of previously published upper endoscopy assessment tools, personal experience, and cognitive interviews with faculty who had specific interests in endoscopy education.

**Table 1.** Skill Assessment in Fellow Endoscopy Training (SAFE-T) upper endoscopy tool

Farthest landmark reached (without any hands-on assistance)
1. Oropharynx
2. Esophagus
3. Stomach
4. Duodenal bulb
5. Second part of the duodenum
6. Other (post-surgical anatomy)
Interventions performed (mark all that apply)
▪ Biopsy
▪ Hemostatic maneuver (clip placement, electrocautery)
▪ APC ablation
▪ Band ligation
▪ Dilation (balloon or Savary)
▪ PEG-tube placement
▪ Polypectomy
▪ Submucosal injection
▪ N/A (no intervention performed)
Case complexity
1. Straightforward (biopsy or no intervention performed)
2. Average (intervention beyond biopsy performed in an accessible location)
3. Challenging (intervention beyond biopsy performed in a difficult location)
Overall performance
1. Beginner (significant hands-on assistance and coaching)
2. Advanced beginner (some hands-on assistance and/or significant coaching)
3. Intermediate (limited hands-on assistance but needs some coaching)
4. Proficient (no hands-on assistance but needs extra time)
5. Superior (able to perform exam independently and efficiently)
For the next case, the fellow should focus on improving this one aspect
▪ Pre-procedure evaluation (indication, co-morbidities, informed consent, etc.)
▪ Patient-discomfort monitoring and management
▪ Esophageal intubation
▪ Gastric retroflexion
▪ Pyloric intubation
▪ Advancement beyond duodenal bulb
▪ Safe endoscopic advancement
▪ Adequately visualized mucosa
▪ Pathology identification
▪ Biopsy technique
▪ Hemostatic technique (clip placement, electrocautery)
▪ APC ablation technique
▪ Band ligation technique
▪ Dilation technique
▪ PEG technique
▪ Polypectomy technique
▪ Submucosal injection technique
▪ N/A (no specific area to improve)

PEG, percutaneous endoscopic gastrostomy; APC, argon plasma coagulation; N/A, not applicable.

### Study participants

All ACGME-accredited general GI fellows in a single GI fellowship program participated in the study. Five fellows each from Years 1–3 of training were included, for a total of 15 fellows. All attending gastroenterologists who supervise fellows performing upper endoscopy were eligible to participate in the study as evaluators. The study was conducted over a 12-month period from July 2018 (start of the academic year) through June 2019. Evaluations were completed at three different clinical sites affiliated with the fellowship program. The study was reviewed by the Institutional Review Board at Partners Healthcare and was given exempt status.

### Data collection

We adapted the SAFE-T upper endoscopy tool into a web-based application that was optimized for use on smartphones as well as computers. Participating faculty downloaded the SAFE-T application onto their smartphones and added the associated icon to their smartphone home screen. Selection of the web-based application icon directly navigated the faculty member to a website that contained the five-part SAFE-T upper endoscopy evaluation form. Each faculty member had a unique login and password to access the application. Completed SAFE-T evaluations were electronically logged and transmitted to a password-protected central repository. Prior to each endoscopy session with a fellow, the web-based application sent an automated email reminder to faculty members to complete SAFE-T evaluations for each observed upper endoscopy.

SAFE-T upper endoscopy evaluations were solicited and completed for each upper endoscopy performed by a fellow from 15 July 2018 to 30 June 2019. Nested within this prospective data collection, an independent reviewer observed an additional 10 upper endoscopies performed by a fellow (from all three years of training) under the guidance of a supervising gastroenterologist in the endoscopy procedure room. The independent reviewer completed the SAFE-T upper endoscopy tool as well as the ACE upper endoscopy form for each of these 10 observed upper endoscopies. The attending gastroenterologist completed the SAFE-T evaluation tool per the normal protocol for these same procedures. The independent reviewer was blinded to the SAFE-T data completed by the attending gastroenterologist for these procedures.

Surveys on the user experience of the SAFE-T tool were sent to participating faculty who completed at least three prior SAFE-T upper endoscopy evaluations by the conclusion of the study period (*n *=* *19).

### Validity evidence

We evaluated the SAFE-T upper endoscopy tool based on Downing’s components of education assessment validity, including content validity, response process, and relationship to other variables [9]. In addition, we assessed the tool’s inter-rater reliability and relationship to a previously validated score.

#### Content validity

Content validity refers to the ability of an instrument to measure what it is intended to do—in this case, the performance of an upper endoscopy. As such, we designed the SAFE-T upper endoscopy form with a team of endoscopy educators and further refined the questions based on cognitive interviews with faculty members. In addition, we followed the framework of the previously validated SAFE-T colonoscopy form and incorporated skills deemed critical to the performance of an upper endoscopy based on review of previously published assessment tools [5–7].

#### Response process

The response process consists of preserving data integrity by eliminating or minimizing sources of error from test administration. Accordingly, we provided frame-of-reference training to each participating faculty member to ensure that they understood each of the five questions and how to submit a SAFE-T evaluation through the web-based application.

We also developed anchors for Questions 3 (case complexity) and 4 (overall performance) to improve the accuracy of responses. To minimize error from data entry, the web-based application automatically uploaded the completed evaluation forms to a central repository. Prior to the start of the study, we conducted a 2-week trial period (July 1st to July 14th, 2018) using the web-based application to address any issues with the electronic logging of data.

#### Relationship to other variables

To assess how the SAFE-T upper endoscopy tool correlated with other relevant variables, we compared the mean SAFE-T overall performance scores across (i) trainee year, (ii) successful vs failed intubation of the second part of the duodenum (D2), and (iii) case complexity. We hypothesized that fellows with more training experience would perform better, cases with successful intubation of D2 would be associated with higher performance scores, and more complex cases would be associated with lower performance scores. Given that we expected a steep learning curve for early fellows, we also compared the mean SAFE-T overall performance score for the first half of the academic year vs the second half for the first-year fellows. We hypothesized that the first-year fellows would perform significantly better with more experience during their first year of training.

#### Inter-rater reliability

For the subset of dual-observed procedures, we calculated the Kappa agreement statistic to compare the SAFE-T overall performance score between the supervising faculty member and the independent reviewer.

#### Relationship to a previously validated score

From the data generated by the independent reviewer in these dual-observed procedures, we calculated the Pearson correlation coefficient (*r*) to assess the correlation between the SAFE-T overall performance score and the overall hands-on score of the ACE upper endoscopy tool.

### Statistical analysis

We calculated means and 95% confidence intervals (CIs) for continuous variables and counts and percentages for categorical variables. We used independent *t*-tests to compare the mean SAFE-T overall performance score between sequential years of training, successful vs failed intubation of D2, and case complexity. We assessed inter-rater reliability with the Kappa agreement statistic. To evaluate the relationship to a previously validated score, we calculated the Pearson correlation coefficient. *P*-values <0.05 were considered significant. All analyses were conducted using JMP Pro version 14 (SAS Institute, Cary, NC).

## Results

### Evaluations

During the study period, 20 faculty used the SAFE-T tool to complete 413 evaluations of upper endoscopies performed by the 15 fellows in the study (Table 2). First-year fellows completed 217 procedures (52.5%), second-year fellows completed 110 procedures (26.6%), and third-year fellows completed 86 procedures (20.8%). Faculty rated the SAFE-T overall performance of the fellows using all five points of the scale: 19 (4.6%) at beginner, 34 (8.2%) at advanced beginner, 68 (16.5%) at intermediate, 173 (41.9%) at proficient, and 119 (28.8%) at superior.

**Table 2.** Skill Assessment in Fellow Endoscopy Training (SAFE-T) overall performance by fellow year

Fellow year	No. of procedures	Beginner	Advanced beginner	Intermediate	Proficient	Superior
First year	217	19 (8.8%)	33 (15.2%)	55 (25.4%)	81 (37.3%)	29 (13.4%)
Second year	110	0 (0.0%)	1 (0.9%)	10 (9.1%)	60 (54.6%)	39 (35.5%)
Third year	86	0 (0.0%)	0 (0.0%)	3 (3.49%)	32 (37.2%)	51 (59.3%)
Total	413	19 (4.6%)	34 (8.2%)	68 (16.5%)	173 (41.9%)	119 (28.8%)

Number of procedures (percentage for each year of training).

### Response to other variables

**Figure 1.**
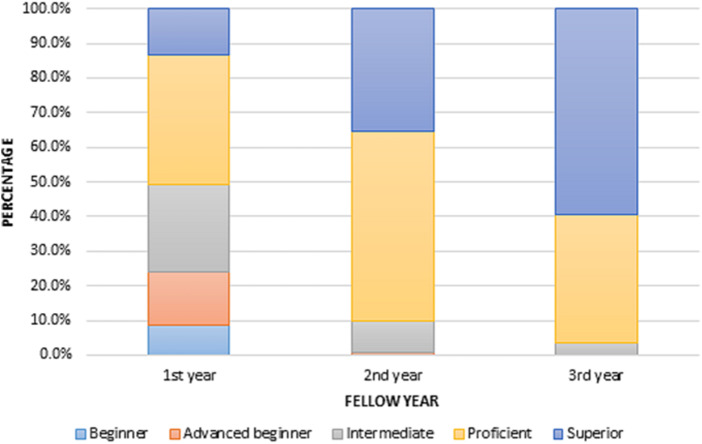
Skill assessment in fellow endoscopy training (SAFE-T) overall performance by fellow year.

The mean SAFE-T overall performance score increased with each additional year of training. The mean SAFE-T overall performance score for first-year fellows was 3.31 (95% CI 3.16–3.47), second-year fellows was 4.25 (95% CI 4.12–4.37), and third-year fellows was 4.56 (95% CI 4.44–4.68). The difference between each subsequent year of training was significant (Year 1 vs Year 2, *P *<* *0.001; Year 2 vs Year 3, *P *<* *0.001). The SAFE-T overall performance score also increased over the length of the study period. First-year fellows had significantly higher SAFE-T overall performance scores in the second half of the academic year compared with the first half (3.96 vs 2.73, *P *<* *0.001). Overall, first-year fellows received the lowest percentage of superior SAFE-T overall performance scores at 13.4% (Table 2 and Figure 1). No second- or third-year fellow received a SAFE-T overall performance score of beginner.

In addition to differentiating fellows based on experience, the mean SAFE-T overall performance score was also significantly higher according to D2 intubation rates (Table 3). Fellows who successfully intubated the second part of the duodenum without hands-on assistance from a supervising faculty had significantly higher mean SAFE-T overall performance scores than those who did not (4.04 vs 2.33, *P *<* *0.001).

**Table 3.** Skill Assessment in Fellow Endoscopy Training (SAFE-T) overall score in response to other variables

Variable	SAFE-T overall score (mean)	*P*-value
Training year		
Year 1 vs Year 2	3.31 vs 4.25	<0.001
Year 2 vs Year 3	4.25 vs 4.56	<0.001
D2 intubation		
Successful vs failed	4.04 vs 2.33	<0.001
Case complexity		
Straightforward vs average	3.97 vs 3.39	<0.001
Average vs challenging	3.39 vs 2.84	0.042

With increasing case complexity, fellows required additional support from their supervising faculty (Table 3). The mean SAFE-T overall performance score for straightforward cases was 3.97 (95% CI 3.87–4.08), average cases was 3.39 (95% CI 3.12–3.66), and challenging cases was 2.84 (95% CI 2.26–3.42). The difference was statistically significant for increasing complexity: straightforward vs average cases (*P *<* *0.001) and average vs challenging cases (*P *=* *0.042).

### Reliability and relationship to a previously validated score

For the 10 dual-observed procedures, the inter-rater reliability for the SAFE-T overall performance score assigned by the supervising faculty member and the independent reviewer was excellent, with a Kappa agreement statistic of 0.815 (*P *=* *0.001). The SAFE-T overall performance score also correlated highly with the previously validated ACE upper endoscopy overall hands-on score (*r *=* *0.76, *P *=* *0.011).

### Survey results

The survey response rate for participating faculty was 74% (14/19). All respondents reported that completing a SAFE-T evaluation using the web-based application took <2 minutes. All respondents also agreed or strongly agreed with the statement “it was easy to complete a SAFE-T evaluation form.” The majority of participating faculty (78.6%) also agreed or strongly agreed that the SAFE-T tool encouraged them to deliver feedback to fellows after supervised upper endoscopies.

Faculty members emphasized the ease of use and role as a reminder to provide feedback to fellows as particular strengths of the SAFE-T tool in the optional comment section of the survey. One faculty member noted that the tool was “easy to use” and a “helpful reminder to give verbal feedback about each procedure to the fellow.” Another faculty member similarly remarked: “The SAFE-T tool is extremely user-friendly and most importantly, it is an excellent reminder to give feedback.”

## Discussion

In this prospective study of ACGME-accredited GI fellows, we developed and provided validity evidence for the SAFE-T upper endoscopy tool as a concise and web-based means of assessing GI fellow performance in upper endoscopy. The SAFE-T overall score differentiated performance based on year of training, D2 intubation, and case complexity. In addition, the SAFE-T overall score showed excellent inter-rater reliability in dual-observed procedures and high correlation with a previously validated tool. Administered electronically via a web application, the SAFE-T tool functions as a point-of-care assessment tool that can readily be used after each supervised upper endoscopy. The vast majority of users reported that the tool was easy to use, took <2 minutes to complete, and encouraged faculty to provide feedback to fellows. By capturing both summative and formative feedback, the SAFE-T upper endoscopy tool may be used by GI fellowship programs to trend fellow performance over time, advise fellows on specific areas to improve, and help faculty tailor teaching to a specific fellow.

We provided validity evidence for the SAFE-T upper endoscopy tool as outlined by Downing, including content validity, response process, and relationship to other variables [9]. We designed the form with a team of endoscopy educators and conducted cognitive interviews with other faculty to ensure content validity. Through frame-of-reference training, anchors for scaled questions, and adapting the tool to a web-based application that automatically uploaded data to a central repository, we met the standards for response process. As we hypothesized, the SAFE-T overall performance score was significantly higher with increased fellow experience and in cases with successful D2 intubation and significantly lower in more challenging cases (relationship to other variables). For additional validity evidence, we demonstrated that the SAFE-T upper endoscopy tool has excellent inter-rater reliability in dual-observed procedures (i.e. two raters evaluating the same event). In addition, we showed that the SAFE-T overall performance score is highly correlated with the previously validated ACE upper endoscopy overall hands-on score.

Given the limited time that attendees have in their busy schedules to complete trainee evaluations, we placed a priority on designing the SAFE-T upper endoscopy tool as a concise means of assessment that was easy to use. We thus limited the evaluation form to five questions and adapted the tool into a web-based application that was optimized for smartphone use and automatically updated all data to a central repository. Our end-of-study survey provided evidence that we met our design goals, as all faculty respondents noted that it took <2* *minutes to complete a SAFE-T upper endoscopy form. Faculty members also all agreed or strongly agreed that it was easy to complete a SAFE-T upper endoscopy form using the web-based application. As such, we believe the SAFE-T upper endoscopy tool can function as a point-of-care assessment tool for real-time assessment in actual clinical practice.

The previously validated ACE and DOPS forms for upper endoscopy are both more comprehensive in their skill assessment than the SAFE-T upper endoscopy form. These two scores require evaluators to rate trainee performance along several cognitive and motor parameters beyond an overall performance score. Of note, the DOPS form does so with 34 distinct ratings of endoscopic skills, whereas the ACE form narrows the skill evaluation to 6 different entities. As a result, these two scoring tools provide more granular data of individual fellow performance in upper endoscopy. However, these data come at the cost of additional questions, which places additional stress on the evaluator and limits their usage for continuous assessment after each procedure. Indeed, the ACE form was completed only after each set of 50 procedures in the validation study [10]. Further, these individual skill scores closely correlate with their corresponding overall performance score, which raises the question of how useful it is to ask the evaluator to complete each skill-specific question. Thus, our approach was to use an overall performance score to obtain a summative assessment of the fellow’s skills and then request the evaluator to choose a single skill to improve on for the following case, which allowed us to obtain similar information but with just two questions. We believe this design choice increased faculty’s willingness to complete SAFE-T evaluations on a more regular basis, as evidenced by the high user satisfaction in our end-of-survey study.

We envision the SAFE-T upper endoscopy tool working in cohort with the ACE upper endoscopy form to provide a continuous and comprehensive assessment of a trainee’s endoscopic skills over the entirety of GI fellowship. Given the ease of use and concise nature of the form, the SAFE-T upper endoscopy tool can be completed after each supervised procedure without significant burden on the attending physician. This continuous assessment produces a wealth of data on the individual fellow as well as the entire fellowship. The resultant learning curves allow for quick identification of fellows in need of additional training and the areas to improve help faculty to tailor their teaching to each individual fellow. As the SAFE-T tool lacks a rating system for individual motor and cognitive skills, the ACE upper endoscopy form can then be used at regular intervals (such as every 50th procedure as in the validation study) for more comprehensive assessment. By taking advantage of the strengths of each tool, this combined approach produces the most complete assessment of a fellow’s endoscopic performance.

The SAFE-T upper endoscopy tool joins the previously validated SAFE-T colonoscopy tool as a more complete means of continuously assessing GI fellow performance in the standard endoscopic procedures outlined by the Gastroenterology Core Curriculum [1]. Both tools differentiate fellow performance by trainee experience and case complexity, have excellent inter-rater reliability, and are highly correlated with a previously validated scoring tool [8]. We thus envision the SAFE-T tools being used in tandem with the continuous assessment of GI fellow endoscopic skills over the course of a 3-year GI fellowship program and in conjunction with interval assessments via the ACE upper endoscopy and colonoscopy forms. Presently, we are in the process of enhancing the web-based application so that the complete SAFE-T tool may be used at any interested fellowship program.

This study does have some important limitations. First, this was a single-center study and thus the results may not be generalizable to other fellowship programs. As such, we intend to conduct a multicenter study of the SAFE-T upper endoscopy tool to provide further validity evidence. Second, we were unable to blind faculty to the fellow they were supervising and thus it is possible that faculty ratings were biased by this knowledge (e.g. fellow’s year of training). However, one can imagine that this would be exceedingly difficult and resource-intensive, and thus was similarly not done in prior studies [5–7, 10]. Third, we were not able to calculate a response rate for the SAFE-T upper endoscopy tool, as there were multiple sites of training—one of which did not have an electronic endoscopy software program to allow case tracking. It is reassuring that all participating faculty agreed or strongly agreed that the tool was easy to use, which increases the likelihood of its use after each procedure. Fourth, we limited the SAFE-T tool to just five questions, which limits the breadth of the tool to rate individual endoscopic skills, and the overall performance score is primarily influenced by motor skills. However, we designed the formative assessment question of the tool to identify a specific area to improve, and included cognitive skills (e.g. pre-procedure evaluation, patient-discomfort monitoring, pathology identification) in this question from which faculty may choose.

In conclusion, the SAFE-T upper endoscopy tool is a valid and concise means of assessing fellow performance in upper endoscopy. Administered via a web-based application, the tool allows for point-of care assessment and joins the previously validated SAFE-T colonoscopy tool to provide an efficient means of evaluating fellow performance in standard endoscopy. When used after each supervised procedure, the SAFE-T tool produces an immense amount of data that allow fellowship programs to track fellow performance over time and identify those who may need additional endoscopic training. Particularly from the formative assessment piece, the tool allows faculty to tailor their teaching to a specific fellow, thereby enriching the educational experience. The SAFE-T tool may also be used to measure the impact of educational interventions on fellow endoscopic performance. As future steps, we aim to make the SAFE-T tool available to any interested fellowship program site and utilize large-scale data to establish performance benchmarks for each fellowship year of training.

## Authors’ contributions

N.L.K. conceived the idea, developed the study design, and wrote the manuscript. G.K. developed the web-based application and oversaw data collection and statistical analysis with N.L.K. K.E.H., M.L.P., and K.J. assisted with study design/implementation and edited the manuscript. J.R.S. provided guidance on the study idea, design, analysis, and manuscript preparation. All authors approved the final manuscript.

## Funding

This work was supported by the Clinical Education Research Scholars Program of the Department of Medicine at Brigham and Women’s Hospital (award recipient—N.L.K.) and the NIH T32 training grant (DK007533035; award recipient—K.E.H.).
